# Moderate Aortic Stenosis and Reduced Left Ventricular Ejection Fraction: Current Evidence and Challenges Ahead

**DOI:** 10.3389/fcvm.2018.00111

**Published:** 2018-08-17

**Authors:** Ernest Spitzer, Ben Ren, Herbert Kroon, Lennart van Gils, Olivier Manintveld, Joost Daemen, Felix Zijlstra, Peter P. de Jaegere, Marcel L. Geleijnse, Nicolas M. Van Mieghem

**Affiliations:** ^1^Department of Cardiology, Thoraxcenter, Erasmus University Medical Center, Rotterdam, Netherlands; ^2^Cardialysis, Clinical Trial Management and Core Laboratories, Rotterdam, Netherlands

**Keywords:** moderate aortic stenosis, left ventricular ejection fraction, transcatheter aortic valve replacement, surgical aortic valve replacement, structural heart disease, TAVR UNLOAD trial

## Abstract

Moderate aortic stenosis (AS) and reduced left ventricular ejection fraction (LVEF) constitute a clinical entity that has been proposed as a therapeutic target for transcatheter aortic valve replacement (TAVR). It is defined by a mean trans-aortic gradient between 20 and 40 mmHg and an aortic valve area between 1.0 and 1.5 cm^2^ in patients with LVEF < 50%. Retrospective data suggests a prevalence of 0.8% among patients referred for echocardiographic assessment. These patients are younger and show a higher frequency of previous myocardial infarction than those with severe AS randomized to TAVR in recent trials. In two retrospective studies including patients with moderate AS and reduced LVEF, a one-year mortality rate of 9 and 32% was reported, the latter in patients treated with medical therapy only during follow-up. Echocardiographic diagnosis of moderate AS poses challenges as current guidelines are directed to determine severe AS, and different presentations of moderate and mild AS have been generally neglected. Thus, the nomenclature would need to be revised and a description of possible scenarios is provided in this review. Dobutamine stress echocardiography and computed tomography are promising complementary tools. Likewise, a standardized clinical pathway is needed, in which a high level of suspicion and a low threshold for referral to a heart valve center is warranted. The Transcatheter Aortic Valve Replacement to UNload the Left ventricle in patients with Advanced heart failure (TAVR UNLOAD) trial (NCT02661451) is exploring whether TAVR would improve outcomes in patients receiving optimal heart failure therapy.

## Introduction

Severe aortic stenosis (AS) is the most common indication for valvular interventions in adults. It affects 3% of patients after 75 years of age (1). Earlier stages of the disease have been typically disregarded as targets for aortic valve replacement (AVR) given an unfavorable risk-benefit balance. Specifically, due to the relatively high rates of peri-procedural death or stroke with AVR, and the lack of evidence of a significant increased risk of events in patients with mild to moderate AS treated medically (2). Better characterization of high risk populations among patients with moderate AS together with advancements of transcatheter aortic valve replacement would identify a niche population potentially benefiting from an earlier intervention (3–5).

In patients with moderate AS and concomitant reduced left ventricular ejection fraction (LVEF < 50%), death or heart failure hospitalization was observed in half of them at 4 years of follow-up (5). It is noteworthy that pathophysiologically LVEF reduction in this population is generally not attributed to AS, but rather to myocardial damage due to ischemic conditions or non-ischemic non-valvular cardiomyopathies. In this setting moderate AS may contribute significantly to the overall ventricular afterload, and contribute to systolic and diastolic dysfunctions and ultimately to a progressive symptomatic status (6, 7). In patients with severe AS and conservative treatment, reduced LVEF has been associated with increased rates of death and heart failure hospitalizations [hazard ratio (HR), 95% confidence interval (CI): 1.82 [1.44–2.28], *p* < 0.001, when compared with LVEF > 70% by the Teichholz or Simpson method] (8). In patients undergoing AVR, reduced EF has also emerged as an independent predictor of mortality at 5 years of follow-up, with an increase of 12% mortality [HR (95% CI) = 0.88 (0.83–0.94), *p* < 0.001] for every 10% decrease in LVEF (9). Among patients with moderate AS and reduced LVEF, male sex, New York Heart Association (NYHA) functional class III and IV, and the peak aortic jet velocity obtained with Doppler ultrasound emerged as independent predictors of worse outcome (5). Advanced heart failure symptoms (NYHA III or IV = 58%, NYHA II = 31%, and NYHA I = 23%, at 2 years) as well as being admitted at the time of diagnosis (60 vs. 34% at 2 years, *p* < 0.001), are associated with increased rate of death, AVR or heart failure hospitalizations (5).

This review summarizes the frequency of concomitant reduced LVEF and moderate AS, its natural history, differential patient characteristics when compared with patients with severe AS, diagnostic challenges, and further discusses the rationale of the ongoing Transcatheter Aortic Valve Replacement to UNload the Left ventricle in patients with ADvanced heart failure (TAVR UNLOAD) trial (NCT02661451) (10).

### Epidemiology and natural history

Reduced LVEF is an established predictor of adverse events including heart failure hospitalizations and death, and its severity determines the treatment alternatives for a patient (11). One to Two Percent of adults live with the diagnosis of heart failure, with a lifetime probability of 1:3 to receive this diagnosis after the age of 55 years (11) Reduced LVEF accompanies this clinical syndrome in approximately 50% of cases. Ambulatory patients with symptomatic reduced LVEF have a one year risk of death of 7% and hospitalizations of 32%; whereas the rates increase to 17 and 44% after one hospitalization (11). Less is known for moderate aortic stenosis, since it is not a target of medical therapy, as several attempts to decelerate the progression of disease have failed (12–14). Moreover, surgical aortic valve replacement (SAVR) is considered only when moderate AS is diagnosed as a bystander in a patient undergoing an open heart surgery for other conditions (15). Consequently, in routine clinical practice, this condition is not prospectively ascertained and streamlined for treatment, leading to difficulties in determining its frequency.

An analysis of the Duke echocardiographic database (*N* = 132,804) showed that 1.2% of patients would qualify for the diagnosis of moderate or severe AS and reduced LVEF, from which 0.8% had moderate and 0.4% had severe AS (16). In an effort of extrapolation, for a center performing 5,000 echocardiograms per year, this would represent the diagnosis of moderate AS and reduced LVEF in 40 patients per year. More importantly, one third of patients with moderate AS included in the aforementioned analysis did not survive more than one year on medical treatment only (16). More optimistic results were observed in a multi-center retrospective analysis, which showed a one-year mortality rate of 9% in patients with moderate AS and reduced LVEF, partially explained by a 13% rate of AVR at one-year (5). Both studies ought to be contrasted with the 1.4% rate of one-year mortality observed in the Simvastatin and Ezetimibe in Aortic Stenosis (SEAS) trial, which randomized 1,873 patients with *mild-to-moderate* AS to an intensive cholesterol lowering strategy (13). Contrary to the previous higher risk cohorts, the SEAS trial included *asymptomatic* patients with no atherosclerotic conditions, diabetes mellitus, or indication of lipid-lowering therapy. Virtually all patients had LVEF above 50 and 30% had an aortic valve area (AVA) above 1.5 cm^2^, consistent with mild AS. This comparison highlights the impact of LVEF and AS severity on clinical outcomes.

The rate of progression of AS has been systemically assessed in the SEAS and other randomized trials investigating the impact of lipid-lowering therapies on the natural history of the disease (12–14). Despite a significant reduction of low-density lipoprotein cholesterol, the disease progression remained comparable among study groups. A meta-analysis of statin trials reported an annual increase in mean trans-aortic gradient of 2.8 ± 3.0 mmHg, and a decrease in aortic valve area of 0.04 ± 0.27 cm^2^ (17). Uncertainty remains on individual factors that may accelerate the progression of the disease (e.g., smoking, serum creatinine), and individual variation has been described with mean rates of progression as high as an annual increase of 7 mmHg in mean gradient and decrease of 0.1 cm^2^ in AVA (18).

Supportive evidence of the detrimental role of increased trans-aortic gradients stems from research on prosthesis-patient mismatch (PPM) after SAVR. PPM is present when the AVA of the inserted valve is too small in relation to the body size of the patient, which generates higher than expected trans-aortic gradients (6). In a cohort of 2,576 patients who underwent SAVR, severe PPM defined as an indexed AVA (AVAi) ≤ 0.65 cm^2^/m^2^, was associated with increased late overall and cardiovascular mortality, after adjustment for other known risk factors (6). Importantly, moderate PPM (AVAi > 0.65 but ≤ 0.85 cm^2^/m^2^) was also associated with increased mortality {HR [95% CI] = 1.21 [1.03–1.41], *p* = 0.01} in patients with reduced LVEF (<50%) only (6). Pathophysiologically, this suggests that patients with impaired systolic function suffer from persistent higher-than-normal afterload. Furthermore, recent evidence suggests that in patients with AS, the decline in LVEF starts before AS is severe and accelerates after AVA reaches 1.2 cm^2^ (19). LVEF < 60% in the presence of moderate AS has been suggested as a predictor of further LVEF deterioration (19).

Hypertension is highly prevalent in patients with moderate AS and reduced LVEF (5). Although not a direct component of the disease, it determines the arterial component of the afterload. The sum of the valvular and arterial components of the afterload represents the valvulo-arterial impedance, (20) which is a strong predictor of mortality in patients in different stages of AS, ranging from moderate to severe asymptomatic patients up to post-TAVR patients (21–23). In elderly patients who typically have a reduced arterial compliance, means to reduce the arterial component of the overall LV afterload with medical therapy i.e., anti-hypertensive drugs, are limited. In this clinical setting AVR for even moderate AS may eventually be the only option to further reduce the valvulo-arterial impedance and improve outcome (10).

### Aortic valve replacement

Current clinical practice guidelines recommend SAVR in patients with moderate AS undergoing CABG or surgery of the ascending aorta or of another valve (class of recommendation IIa and a level of evidence C) (15, 24) Moreover, Patients undergoing CABG before 70 years of age with a peak gradient above 30 mmHg and a documented yearly progression of 5 mmHg, may benefit from SAVR (2). These represent the only two mentions of moderate aortic stenosis in the ESC/EACTS guidelines for management of valvular heart disease, and are largely supported by retrospective data (15). Such interventions are referred to as “prophylactic SAVR,” aiming to avoid a second open-heart procedure. A post-CABG SAVR procedure is exposed to the risk of damaging patent grafts including the internal mammary arteries; is technically challenging due to calcified aortic arches and scarring of the mediastinum; and procedural mortality has been reported in up to 16% of patients (25, 26) In patients with prior CABG that require AVR, TAVR is progressively replacing SAVR (26). In a propensity-matched analysis including 3,880 record in each group, TAVR and SAVR showed similar in-hospital mortality (2.3 vs. 2.4%, *p* = 0.71) but TAVR was associated with lower incidence of procedural complications including myocardial infarction (1.5% vs. 3.4%, *p* < 0.001), stroke (1.4 vs. 2.7%, *p* < 0.001), bleeding (10.6 vs. 24.6%, *p* < 0.001), and acute kidney injury (16.2 vs. 19.3%, *p* < 0.001). Consequently, the rationale of exposing the patient to a prophylactic SAVR in order to avoid a second open-heart surgery is currently challenged (26).

The concept of “therapeutic” TAVR in patients with moderate AS is evolving (10). In a retrospective analysis of 1,090 patients with moderate AS and reduced LVEF, SAVR within 90 days vs. no intervention, was associated with a 41% reduction of all-cause mortality after a median follow-up time of 1.2 years (16). Moreover, this benefit remained in a sub-group of patients with reduced LVEF without coronary artery disease (16).

### Patient characteristics

Patients with moderate AS and reduced LVEF are younger and show a higher frequency of prior acute myocardial infarction than patients with severe AS typically included in randomized trials (see Table 1) (3, 5, 16, 29, 32). Importantly, prospective randomized trials comparing TAVR vs. SAVR in bicuspid aortic valve disease may provide guidance on the preferred approach for such sub-group, which has been largely excluded from TAVR studies. Post-TAVR incidence of moderate to severe paravalvular leak and new pacemaker implantation has been more frequently observed in patients with bicuspid anatomy (33). Furthermore, it remains unclear whether there is a preferred device, being a balloon-expandable, self-expandable, or mechanically-expanded transcatheter heart valve, when treating patients in potential need for a re-do procedure due to an expected longer life expectancy (34, 35).

**Table 1:** Clinical characteristics in patients with moderate or severe aortic stenosis.

	**Moderate AS and reduced LVEF**	**Moderate AS and reduced LVEF**	**Severe AS and reduced LVEF**	**Severe AS inoperable**	**Severe AS extreme risk**	**Severe AS high risk**	**Severe AS high risk**	**Severe AS intermediate risk**	**Severe AS intermediate risk**	**Severe AS all-comers**
Age, years	73	73	76	83	83	84	83	82	80	79
Female gender, %	25	31	40	54	52	42	46	46	42	46
Hypertension, %	74	68	67	NA	90	NA	95	NA	93	71
Diabetes, %	38	39	32	NA	42	NA	35	38	34	18
Peripheral vascular disease, %	20	17	14	30	35	43	42	28	31	4
Cerebrovascular disease, %	43	23	21	27	23	29	26	32	13	17
Atrial fibrillation, %	NA	32	32	33	47	41	41	31	28	28
Chronic pulmonary disease, %	25	11	6	NA	NA	43	NA	32	NA	12
Prior myocardial infarction, %	52	39	35	19	31	27	26	18	14	6
Prior PCI, %	36	13	10	31	37	34	34	27	21	8
Prior CABG, %	28	22	19	37	40	43	30	24	16	NA
Ischemic heart disease, %	48	63	64	68	82	75	75	69	63	NA
Patients analyzed	305	403	935	179^*^	489	348^*^	394^*^	1011^*^	879^*^	145^*^
References	van Gils et al. (5)	Samad et al. (16)	Samad et al. (16)	Leon et al. (27)	Popma et al. (28)	Smith et al. (29)	Adams et al. (30)	Leon et al. (3)	Reardon et al. (31)	Thyregod et al. (32)
Study type	Retrospective	Retrospective	Retrospective	RCT	Prospective	RCT	RCT	RCT	RCT	RCT

* *Includes subjects randomized to transcatheter aortic valve replacement. AS, aortic stenosis; LVEF, left ventricular ejection fraction; PCI, percutaneous coronary intervention; CABG, coronary artery bypass graft surgery; NA, not available; RCT, randomized controlled trial*.

### Challenges

Three main challenges exist for promoting “therapeutic” TAVR in patients with moderate AS and reduced LVEF: (1) better understanding of the echocardiographic diagnosis of moderate AS, (2) defining a clinical pathway to identify patients, and (3) evidence from randomized trials supporting TAVR in this population.

### Echocardiographic diagnosis of moderate aortic stenosis

Moderate AS is characterized by a mean trans-aortic gradient between 20 and 40 mmHg and an AVA between 1.0 and 1.5 cm^2^. Other findings include a peak velocity of the trans-aortic flow between 3.0 and 4.0 m/s, a velocity ratio between 0.25 and 0.50, and an indexed AVA by body surface area between 0.60 and 0.85 cm^2^/m^2^ (36). When findings are concordant (e.g., mean gradient between 20 and 40 mmHg and AVA between 1.0 and 1.5 cm^2^), the diagnosis is clear-cut. However, discordant findings are frequently observed (e.g., AVA < 1.0 cm^2^ and mean gradient between 20 and 40 mmHg; or, AVA between 1.0 and 1.5 cm^2^ and mean gradient < 20 mmHg), especially in the context of reduced LVEF.

**Figure 1:**
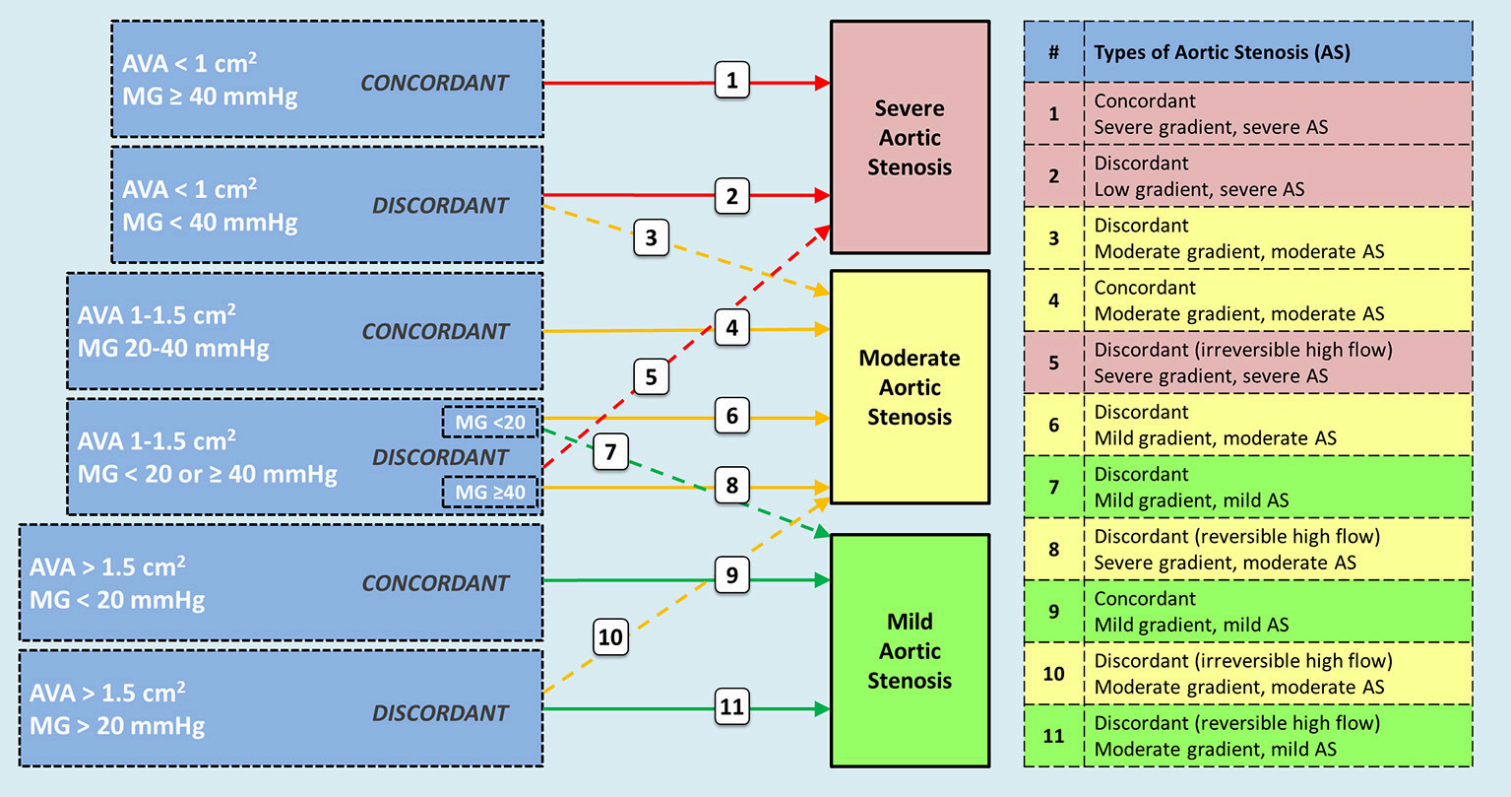
Possible scenarios observed in patients with aortic stenosis including sub-groups according to ejection fraction and flow patterns. Concordant findings between aortic valve area and transvalvular gradient do not pose diagnostic challenges. However, discordant findings require additional tests to define the appropriate category (e.g., dobutamine stress echo, CT-derived aortic valve calcium score, 3D-echo or CT-derived left ventricular outflow tract area). Interpretation of findings are better stablished for categories 2 and 3 (true severe vs. pseudosevere aortic stenosis); however, knowledge is evolving for categories 6 and 7. Patient with high transvalvular flow should be classified after determining if the mechanism of high flow is reversible (e.g., fever, anemia) or irreversible (e.g., concomitant aortic regurgitation). Categories 8 and 11 correspond to reversible causes, and 5 and 10 to irreversible causes. Patients with aortic stenosis can be further categorized based on ejection fraction and flow status. AVA = aortic valve area; MG = mean gradient.

In Figure 1 we summarize the different scenarios that could be observed when evaluating a patient with AS. Our understanding of severe AS has increased significantly in the last two decades, and the most recent classification of patients includes sub-groups based on gradient (low-gradient: mean gradient < 40), ejection fraction (abnormal <50%) and flow status (low flow < 35 ml/m^2^) (36). All flow-gradient patterns, stratified by LVEF, have been reported in patients with severe AS (36–38). When findings for severe AS are discordant in patients with reduced LVEF (AVA < 1 cm^2^ and mean gradient < 40 mmHg) patients are assessed with a low-dose dobutamine stress echocardiogram (DSE) to differentiate among “true severe” AS vs. “pseudo-severe” AS (e.g., moderate AS) (36). Likewise, the difference between mild AS and moderate AS would need to be established through a DSE if therapeutically relevant. For descriptive purposes, Figure 1 creates AS groups based on the following parameters: (1) AS severity is concordant if the AVA and the mean gradient match the same category (i.e., mild, moderate or severe), and if not, AS severity is defined as discordant; (2) In this description, the gradient is not defined as “high” or “low” gradient, but rather in relationship with the category it applies (i.e., mild, moderate, or severe); (3) sub-groups can be further classed based on LVEF (above or below 50%) and flow status (above or below 35 ml/m^2^). With this description, “pseudo-severe AS” would be referred to as “discordant moderate-gradient moderate AS.” It is “discordant” because initially the AVA would be compatible with severe AS (<1 cm^2^) but the gradient with moderate AS (<40 mmHg). All initially discordant cases need further evaluation in order to be properly classified, especially with concomitant reduced LVEF: (1) make sure that there are no technical pitfalls in the measurement of the mean gradient (e.g., misalignment of the Doppler signal with the direction of the flow, inadvertent recording of mitral regurgitation) or the AVA (e.g., underestimation of the left ventricular outflow tract diameter); (2) if LVEF is the factor that could potentially influence the gradient and aortic valve opening, a low-dose DSE is indicated, starting at 2.5 or 5 μg/kg/min with a progressive increase in the infusion every 3–5 min to a maximum dose of 10–20 μg/kg/min; (36) (3) in the absence of contractile or flow-reserve, the computed tomography aortic valve calcium score helps to determine the likelihood of having severe AS (e.g., likely if ≥ 2000 in men, and if ≥ 1200 in women) (36). Currently, there is insufficient data to differentiate mild from moderate AS based on calcium score; (4) increased gradient due to high trans-valvular flow should be considered as an option in patients with discordant findings. When causes are reversible (e.g., fever or anemia), patients should be reassessed after correcting the causes. When causes are irreversible (e.g., significant aortic regurgitation), patients should be categorized according to the gradient severity and treatment should be offered accordingly.

These concepts open a new era in the diagnosis of aortic stenosis, and registry data as well as retrospective analysis may help us clarify the need for an updated nomenclature, which would require a joint effort of cardiology societies.

### Clinical pathway for moderate aortic stenosis and reduced LVEF

Offering TAVR to patients with moderate AS and reduced LVEF would be a change of a clinical paradigm. Due to the currently non-existing treatment alternatives apart from established heart failure therapies, these patients do not fall into any specific clinical pathway. If these patients were to be offered TAVR, imaging will play a pivotal role. Patients with moderate AS and reduced LVEF may be referred within the same institution or through other referral institutions due to symptoms, physical exam (e.g., systolic murmur), or screening echocardiogram. Severity of AS in the context of reduced LVEF may need to be confirmed with a DSE (if initial findings are discordant). Moreover, once the diagnosis is confirmed technical plausibility of TAVR needs to be assessed by means of a pre-TAVR multi-slice computed tomography. Current evidence supports the use of transfemoral over non-transfemoral access for TAVR, given a lower rate of procedural complications (e.g., acute kidney injury, need for renal replacement therapy) and lower 1 year mortality (39, 40). Thus, a transfemoral approach should be considered as first option for this high risk population, commonly avoiding the need of general anesthesia. Most patients would have coronary artery disease, and a coronary angiogram would need to be included in the clinical work-up.

It is essential to refer potential candidates to experienced heart valve centers (15). A high grade of suspicion and low threshold for referral would be required from non-interventional cardiologists and other specialties. Moreover, a systematic echocardiographic assessment of the AVA in all patients with at least mild AS gradient, would potentially help to identify patients with “discordant mild-gradient moderate AS,” which would otherwise have been missed. Nevertheless, current evidence recommends watchful waiting and periodic echocardiographic follow-up in patients with moderate AS and reduced LVEF. This approach may significantly change if results of ongoing trials prove to be clinically meaningful.

### TAVR UNLOAD trial

The single most important requirement to promote TAVR as a therapeutic option in patients with moderate AS and reduced LVEF is to create confirmatory prospective evidence that this intervention is clinically meaningful (5, 6, 16). The Transcatheter Aortic Valve Replacement to UNload the Left ventricle in patients with Advanced heart failure (TAVR UNLOAD) trial (NCT02661451) is an international, multicenter, randomized, open-label, clinical trial comparing TAVR with the Edwards SAPIEN 3 Transcatheter Heart Valve in addition to optimal heart failure therapy (OHFT) vs. OHFT alone in patients with moderate AS and reduced LVEF (10). This trial is currently enrolling patients in The Netherlands, Canada, and the United States of America. Screening of patients includes echocardiographic eligibility assessment by an independent Core Laboratory, which centrally confirms the presence of moderate AS and reduced LVEF. Assessment may include a DSE. Importantly, written confirmation of OHFT is provided by a local heart failure specialist. Clinical, imaging, and procedural eligibility are confirmed by a Central Screening Committee before randomization. The primary endpoint, defined as the hierarchical occurrence of all-cause death, disabling stroke, hospitalizations related to HF, symptomatic aortic valve disease or non-disabling stroke, and the change in the Kansas City Cardiomyopathy Questionnaire, will be analyzed at 1 year. Changes in heart failure pharmacologic and device therapies will be monitored. Moreover, echocardiographic endpoints will be assessed up to 2 years. Findings may have a significant impact on the way we diagnose, refer and manage patients with AS.

## Conclusion

Patients with moderate AS and reduced LVEF are exposed to a significant risk of clinical events including death. Indirect evidence suggests that aortic valve replacement may offer a clinically meaningful benefit. Incorporating this entity as a therapeutic target requires re-assessment of how we diagnose AS and improved strategies of referral. The TAVR UNLOAD trial is investigating whether TAVR could improve clinical outcomes including quality of life in this high risk population.

## Author contributions

ES and NVM conceived the manuscript. ES wrote the first draft in collaboration with BR, MG, and NVM. All co-authors critically reviewed and approved the final manuscript.

### Conflict of interest statement

NVM has received institutional grants from Edwards Lifesciences, Medtronic, Boston Scientific and Abbott. ES has received speaker fees from Edwards Lifesciences. The remaining authors declare that the research was conducted in the absence of any commercial or financial relationships that could be construed as a potential conflict of interest.
